# Prevalence of Anemia and Associated Factors among Newly Diagnosed Patients with Solid Malignancy at Tikur Anbessa Specialized Hospital, Radiotherapy Center, Addis Ababa, Ethiopia

**DOI:** 10.1155/2019/8279789

**Published:** 2019-10-20

**Authors:** Edosa Kifle, Mintewab Hussein, Jemal Alemu, Wondemagegnhu Tigeneh

**Affiliations:** ^1^Wollega University, Institute of Health Science, Department of Medical Laboratory Science, P.O. Box: 395, Nekemte, Ethiopia; ^2^Addis Ababa University, School of allied Health Science, Department of Medical Laboratory Science, Addis Ababa, Ethiopia; ^3^Addis Ababa University, Tikur Anbessa Specialized Hospital, Radiotherapy Center, Addis Ababa, Ethiopia

## Abstract

**Background:**

Anemia is a common finding in cancer, which is caused by many factors. It is a major cause of morbidity in cancer patients, worsens disease status and impairs treatment outcome; however, little is known about the prevalence of anemia and associated factors among cancer patients during diagnosis in developing countries like Ethiopia. In response to this, we have conducted research with the aim of assessing the prevalence of anemia and associated factors among newly diagnosed patients with solid malignancy at Tikur Anbessa Specialized Hospital (TASH), Radiotherapy center, Addis Ababa, Ethiopia.

**Methods:**

Descriptive cross-sectional study was conducted from April to May 2014. A total of 422 newly diagnosed patients with solid malignancy attending Radiotherapy center, TASH were enrolled to assess anemia prevalence and associated factors. Data were coded, entered and analyzed using SPSS version16. Using logistic regression, chi squares, Odds ratio and 95% confidence intervals were computed to measure strength of association between variables. *p*-value < 0.05 was taken as statistically significant.

**Result:**

Out of 422 respondents, 285 (68%) were females and 153 (36%) of respondents fell into 35–49 age group with age range between 18 and 80 years and the median age of 45. Magnitude of solid cancers was gynecologic (28.9%), breast (22.7%), nasopharyngeal carcinoma (NPC) (7.6%), colorectal (7.1%), sarcoma (6.9%), head and neck (4.5%), thyroid (3.3%), hepatoma (1.9%), and others (17.1%). The overall prevalence of anemia across different tumor was 23% and higher anemia prevalence was noted in gynecologic (37.7%) and colorectal carcinomas (26.7%). The majority of the anemic patients (68%) remained untreated for anemia. The mean trigger hemoglobin for transfusion was 7.7 g/dl. About 83.5% of anemia was mild to moderate type. Performance status (AOR = 3.344; 95% CI 1.410–7.927) and bleeding history (AOR = 3.628; 95% CI 1.800–7.314) showed statistically significant association with occurrence of anemia with *p*-value < 0.05.

**Conclusion:**

Among solid cancers, gynecologic cancer remained the dominant one. Anemia prevalence was 23% in general, in which gynecologic and colorectal cancers were more prevalent. ECOG performance status and bleeding history showed a statistically significant association with the occurrence of anemia.

## 1. Background

Anemia is a condition that develops when there is no sufficient healthy red blood cell, which is characterized either by a reduction in HGB, RBC or HCT count below normal levels [1–4]. As per the National Comprehensive Cancer Network (NCCN) guideline, anemia is defined as HGB ≤ 11 g/dl or ≥ 2 g/dl below the baseline. Cancer is one of the most frequent conditions associated with anemia of chronic disease; meantime, anemia is a common complication of cancer [5]. The estimated prevalence of anemia varies ranging from 30% to 90% of cancer patients during the course of their diseases [2, 5, 6].

Cancer-related anemia may occur as a direct effect of neoplasm, by the sensitization of the immune system, or as a result of the cancer treatment whether surgery, radiotherapy or chemotherapy [7, 8]. Cancer itself can directly cause or exacerbate anemia either by suppressing hematopoiesis through bone marrow infiltration or production of cytokines that lead to iron sequestration, inhibit release and synthesis of endogenous erythropoietin, reduce the response of erythroid progenitor cells to erythropoietin, which ultimately impair erythropoiesis [9–11].

Tumor cells are known to produce cytokines such as IL-1, interferon-*γ*, Il-6 and TNF-*α* that may be able to decrease HGB levels by hemolysis, suppression of erythropoiesis, and impairment of erythropoietin response of erythroid medullary precursors [8, 12, 13].

Blood loss can result from hemorrhage of the tumor itself (e.g., hepatoma, gastrointestinal, bladder, gynecologic) [10, 14] and organ damage can further exacerbate anemia from cancer.

Anemia is a major contributing factor to tumor hypoxia, which worsens the results of radiotherapy and chemotherapy, contributes to the progression of cancer and prolongs the duration of the treatment time and lessens the survival rate [12, 15, 16, 18]. Furthermore, anemia causes energy imbalance and emotional distress (fatigue) [21].

## 2. Methods

### 2.1. Study Setting and Study Population

The study was undertaken at Tikur Anbessa Specialized Hospital from April to May 2014, Addis Ababa, Ethiopia. Among the treatment-naïve newly diagnosed confirmed solid cancer patients visited the radiotherapy center during the study period, 422 study participants were determined with the help of a single population proportion. Patients on follow up for chemotherapy or radiotherapy or surgery, with confirmed hematologic malignancy, who took anemia correction treatment, were excluded from the study.

### 2.2. Data Collection

Data on the socio-demographic and clinical characteristics of the study participants were collected using a pretested structured questionnaire by interview and review of medical records. About 4 ml of venous blood was collected by an experienced laboratory technologist from each study participant for HGB, MCV, MCH, and MCHC analyses. These parameters were determined using the hematology analyzer Cell-Dyn 1800 (Abbott Laboratories Diagnostics Division, USA). To ensure the quality of data, pre-testing was done on patients being managed at the radiotherapy center before the study. The performance of the hematology analyzer was controlled by running quality control material alongside the study participant's sample. In addition, all flagged specimen was subjected to the manual differential to confirm the results.

### 2.3. Statistical Analysis

The data were cleaned, edited, checked for completeness, processed, and then entered into Epi Info version 3.5.3 and transported to SPSS version 16 statistical software. Chi-square and Odd's ratio were computed to see association and relationships between prevalence and severity of anemia with risk factors. *p*-value < 0.05 was considered as statistically significant.

### 2.4. Ethical Consideration

Ethical clearance and approval were obtained from Departmental Research and Ethical Review Committee of Addis Ababa University, Department of Medical Laboratory Science. Permission for the conduct of the study was also obtained from the University Hospital. After study participants were informed about the objectives of the study and assuring confidentiality of their data, written informed consent was taken from all the participants.

## 3. Results

### 3.1. Distribution of Socio-Demographic Factors

Out of 422 respondents, 278 (66%) were females and the rest 144 (34%) were males. From the age category, the majority of respondents, 153 (36%) fell into 35–49 age group with age range between 18–80 years and a median age of 45. Two hundred twenty-seven (53.8%) and three hundred twenty-one (76.1%) respondents were urban dwellers and married, respectively. More than half of the respondents were illiterate and 156 (37.0%) patients were housewife (Table 1).

### 3.2. Prevalence of Anemia

A total number of 422 cancer patients, who were first diagnosed at TASH, Radiotherapy center of Addis Ababa University during April–May 2014 were searched and enrolled for analysis. The types of cancer included were gynecologic (122 cases), Breast (96 cases), Nasopharyngeal (32 cases), colorectal (30 cases), Soft tissue sarcoma (29 cases), head and neck cancers (19 cases), thyroid (14 cases), hepatoma (8 cases), and other cancers (72 cases) (Table 2).

The hemoglobin level for the whole patients ranged from 4.6 g/dl to 18.9 g/dl with a mean of 12.6 ± 2.3 (mean ± SD). The mean hemoglobin for male patients was 13.3 ± 2.5 and for female patients, 12.2 ± 2.1 g/dl. More than 1/3 of the anemic patients (68%) remained untreated for anemia. Only 25.8% and 6.2 % of anemic patients were treated with transfusion and iron respectively. The mean trigger hemoglobin for transfusion was 7.7 ± 1.7 (mean, SD) g/dl.

**Figure 1:**
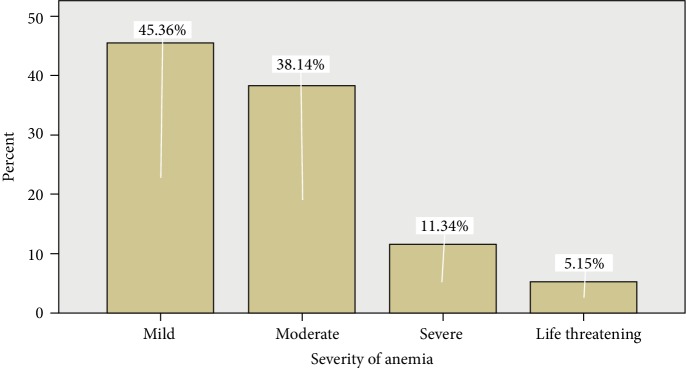
Distribution of severity of anemia among anemic respondents at TASH, Radiotherapy center, Addis Ababa from April to May 2014 (*n* = 97) [Anemia grading: grade 1 or mild = 10−lower limit of normal g/dl; grade 2 or moderate = 8−10 g/dl; grade 3 or severe = 6.5−8 g/dl; grade 4 or life-threatening = < 6.5 g/dl].

Anemia was diagnosed in 97 of the 422 patients (23%) and mean concentration (± SD) of HGB was 13.5 ± 1.5 g/dl in 325 non anemic patients while that was 9.4 g/L ± 1.6 g/dl in 97 anemic patients. Overall, the prevalence of anemia at diagnosis of cancers was 23.0% in unclassified cancers, and higher anemia prevalence was noted in gynecologic (37.7%) and colorectal cancers (26.7%) (Table 2) Majority of the anemia (83.5%) was mild-moderate type whereas 11.3% and 5.2% were severe and life-threatening type (Figure 1).

Among the anemic solid cancer patients, anemia was morphologically categorized based on MCV and MCHC values using the cut off values in Table 3.

**Figure 2:**
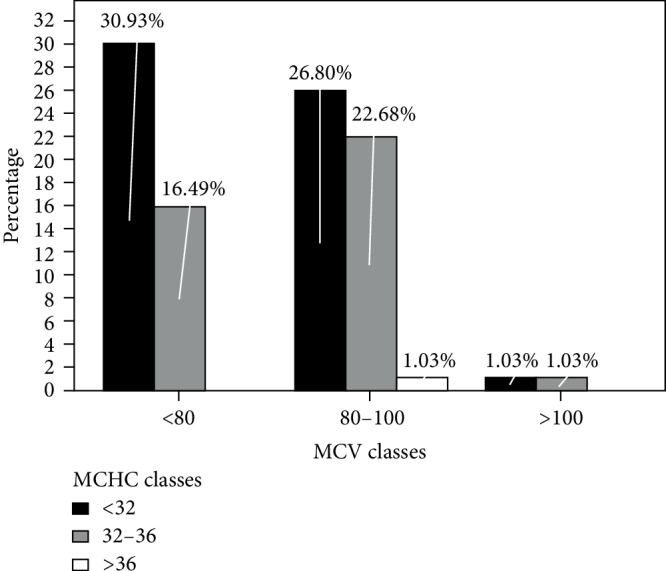
Morphological classification of anemia among anemic patients with solid tumor attending TASH, Radiotherapy center, Addis Ababa from April to May 2014 (*n* = 97).

Accordingly, from the total anemic patients, half of anemia (50.5%) was normocytic anemia, in which normocytic normochromic is 22.7% and normocytic hypochromic is 26.8%, and others were (47.4%) microcytic anemia, in which microcytic hypochromic is 30.9% and microcytic normochromic is 16.5%), and macrocytic anemia (2.1%) (Figure 2).

### 3.3. Risk Factors Associated with the Severity of Anemia

Patients with bleeding history suffered more severe anemia as compared to a patient without bleeding history with *p*-value < 0.05. Nevertheless, there was no statistically significant difference found in gender and age group among the severity of anemia (Table 4).

### 3.4. Risk Factors for the Occurrence of Anemia

In Bivariate analysis, the occurrence of anemia showed statistically significant association with gender, age group, bleeding history, tumor type, tumor stage and ECOG performance status with *p*-value < 0.05.

When multivariate analysis was computed for these variables, a statistically significant association was noted only between the occurrence of anemia with bleeding history and ECOG performance status while considering other variables as confounders.

Patients complained of bleeding history were 4 times more likely to develop anemia than those lacking bleeding history (AOR = 3.628; 95% CI 1.800–7.314).

Patients with ECOG performance status of 3 were 3 times more prone to develop anemia than patients of 0 ECOG performance score (AOR = 3.344; 95% CI 1.410–7.927) (Table 5).

## 4. Discussion

Anemia in cancer patients observed as a result of the malignancy itself, anti-cancer treatment, blood losses, nutritional deficiencies, hemolysis, endocrine disorders, or inflammatory cytokines associated with chronic diseases. In our data, 422 treatment-naïve, newly diagnosed solid cancer patients in TASH, Radiotherapy center were included for this analysis. According to this study, the overall prevalence of anemia across different tumor was 23%, which is higher than the study conducted in China, 18.98% [19]. However, our finding is lower than the reports made by other researchers that showed 39.3%, 35%, 41%, 54.4%, 54.7%, and 55.7% in Europe, Australia, USA, Thailand, India, and Belgium, respectively [11, 17, 22, 24–26]. The low prevalence in our study is because of the difference in definition of anemia, study population and survey period.

As our report revealed, the most common cases noted were gynecologic issues (28.9%) followed by breast carcinoma (22.7%), our results are similar to those of a study conducted in Thailand, where gynecologic (30.6%) and breast cancers (26.2%) scored the first two ranks among the observed tumor types [25].

The prevalence of anemia was varied by tumor type. Our study demonstrated that 37.7% and 26.7% of gynecologic and colorectal cancer patients were anemic, respectively. This finding is lower with the report in Europe and Australia, which revealed 49.1% and 65% of gynecologic cancer patients were anemic at enrollment, respectively [20, 23]. This may be attributed to the difference in the definition of anemia and study design used.

Females and elderly patients with ≥ 65 years ranked higher anemia prevalence rates. We found a similar result in China, Sudan, Belgium [19, 21, 26]. In our survey, females are more anemic than males because of the fact that the majority of the cancer cases noted are gynecologic and the majority of gynecologic patients (53.7%) complained of bleeding history. The primary possible reason for the higher anemia proportion in elder than younger patients is due to the fact that as one gets older, there is a physiological change. As a result of this, for example, there is a decline in hematopoietic stem cell reserves and proliferation capacity, which leads to suppression of erythropoiesis.

Our study showed two factors were significantly associated with the occurrence of anemia, namely ECOG Performance score and bleeding history. Patients with ECOG Performance status 3 were 3.344 times at a higher risk of developing anemia than patients of 0 ECOG performance score, which is in agreement with the study done in the USA [6].

Our study also indicated that patients with bleeding history were 4 times at a higher risk of developing anemia than those patients lacking bleeding history. This finding is similar to reports made in India and China [17, 19] which revealed that bleeding from the tumor were major contributing factors for the occurrence of anemia in patients with solid malignancies. In our study, a majority of the anemic gynecologic patients were complaining of bleeding history 34/97 (35%), which is a contributing factor for the higher (37.7%) anemia prevalence in gynecology among observed tumor types.

In our data, the majority of the anemia (83.5%) was mild to moderate type. The mean trigger hemoglobin level for initiating transfusion in our data was 7.7 g/dl, which is lower as compared to reports made in ACAS (9.5 g/dl), Thailand (8.6 g/dl), Thailand (9.3 g/dl) and ECAS (9.7 g/dl) [12, 22, 23, 25]. The possible justifications for the low mean trigger hemoglobin level in our study are due to variation among Doctors` decision in initiating anemia supportive treatment and also as a result of the high frequency of Grade 3 anemia when compared to other findings.

Regarding the anemia treatment patterns, our data showed that anemia was treated in 32% of patients with anemia. Our result was similar to the reports made in ECAS [22] and ACAS [23] in which 38.9% and 41% of patients with anemia were treated for their anemia before commencing anti-tumor agents, respectively, whereas it was higher compared to that of Thailand [25], in which 22.3% of patients with anemia got anemia correction treatment prior to commencing anti-cancer treatment. The most commonly used supportive treatment for anemia correction was blood transfusion (25.8%), which is in agreement with that of Thailand and ACAS (36%) [22, 23].

Anemia prevalence was also varied by tumor types. Higher anemia prevalence was noted in gynecologic and colorectal carcinomas, 37.7% and 26.7%, respectively. The possible underlying justifications for this finding are because of the disorder of digestive function, unperceived and long term bleeding occurred in the colorectal tumor [19]. The other possible reason for gynecologic patients is all of them are females in gender and several of them also complained of vaginal bleeding history. This is in agreement with the reports made in China, colorectal (23.13%) scoring the 2nd rank followed by gastric (38.02%) and in Australia, where gynecologic (65%) was followed by urogenital (50%) [19, 23].

Our data also showed that bleeding history was found to be a risk factor for severity of anemia with *p*-value < 0.05. This finding is similar to a study done in China showing that patients with bleeding were more likely to have more severe anemia as compared to patients without bleeding [19]. Gender and age category did not show any evidence of association with severity of anemia.

The majority of the anemia in our study was hypochromic (59%), which was different from the study done in China [19], in which 68.6% were normocytic. The underlying possible justification for this variation may be due to the difference in the study population and study period whereas the proportion of macrocytic anemia (1.9%) was similar to our result (2.1%).

Our study demonstrated that ECOG performance status and bleeding history showed a statistically significant association with the prevalence of anemia. This finding is similar to the result reported in China [19] (OR = 1.78, 95% CI; 1.29–2.45) and India [17], in which bleeding from tumor showed statistically significant association with the occurrence of anemia.

## 5. Conclusion

In our study, the overall prevalence of anemia across different tumors is 23%. From the tumor types, gynecologic and colorectal scored higher anemia prevalence compared to others, which are 37.7% and 26.7%, respectively. Female and ≥ 65 aged patients showed a higher frequency of anemia when compared with male and < 65 aged patients. Our study also revealed that ECOG PS and bleeding history indicated statistically significant association (*p* < 0.05) with the occurrence and severity of anemia. The mean hemoglobin for initiating transfusion was 7.7 g/dl.

## Figures and Tables

**Table 1:** Distribution of socio-demographic factors of the respondents at Radiotherapy center, TASH, Addis Ababa, 2014 (*n* = 422).

Variables	Frequency	Percentage
*Age, in years*		
18–34	78	18.5
35–49	153	36.3
50–64	135	32.0
>65	56	13.3
*Sex*		
Male	144	34.1
Female	278	65.9
*Residence*		
Rural	195	46.2
Urban	227	53.8
*Marital status*		
Single	51	12.1
Married	321	76.1
Divorced	15	3.6
Widowed	35	8.3
*Level of education*		
Literate	186	44.1
Illiterate	236	55.9
*Occupation status*		
Employed	85	20.1
Merchant	48	11.4
Farmer	94	22.3
Student	17	4.0
Day laborer	22	5.2
House wife	156	37.0

**Table 2:** Prevalence of anemia among associated factors in newly diagnosed solid cancers at TASH, Addis Ababa, Ethiopia, from April to May 2014 (*n* = 422).

Factors	No (%)	HGB range, in g/dl	Mean HGB ± SD, in g/dl	Percentage with HGB ≤ 11 g/dl
*Sex*				
Male	144 (34.1)	4.8–18.9	13.28 ± 2.47	25 (17.4)
Female	278 (65.9)	4.6–18.7	12.18 ± 2.1	72 (25.9)
*Age categories*				
18–34	78 (18.5)	7.8–18.4	13.43 ± 2.15	10 (12.8)
35–49	153 (36.3)	4.6–18.7	12.45 ± 2.4	40 (26.1)
50–64	135 (32.0)	5.1–18.9	12.45 ± 2.2	30 (22.2)
>65	56 (13.3)	6.2–14.8	11.89 ± 2.11	17 (30.4)
*Tumor types*				
Gynecology	122 (28.9)	4.6–16.2	11.45 ± 2.32	46 (37.7)
Breast	96 (22.7)	8.2–16.4	12.95 ± 1.52	14 (14.6)
Colorectal	30 (7.1)	4.8–18.3	12.34 ± 2.83	8 (26.7)
NPC	32 (7.6)	8.6–16.4	12.49 ± 1.95	8 (25.0)
Sarcoma	29 (6.9)	7.2–18.4	13.09 ± 2.46	5 (17.2)
Head and neck	19 (4.5)	7.8–15.9	13.29 ± 2.29	3 (15.8)
Thyroid	14 (3.3)	10.9–15.7	13.19 ± 1.70	2 (14.3)
Hepatoma	8 (1.9)	10.5–14.7	13.16 ± 1.19	1 (12.5)
Others	72 (17.1)	4.8–18.9	13.43 ± 2.46	10 (13.9)
*Tumor stages*				
Stage I	54 (12.8)	4.8–18.7	13.35 ± 2.35	7(13.0)
Stage II	129 (30.6)	4.8–18.3	12.53 ± 2.30	32 (24.8)
Stage III	174 (41.2)	4.6–18.9	12.15 ± 2.39	51 (29.3)
Stage IV	65 (15.4)	8.3–17.7	13.02 ± 1.66	7 (10.8)
*ECOG PS*				
Grade 0	78 (18.5)	7.9–18.7	13.04 ± 2.09	12 (15.4)
Grade 1	154 (36.5)	6.9–18.9	12.77 ± 2.19	33 (21.4)
Grade 2	87 (20.6)	4.6–17.7	11.95 ± 2.56	26 (29.9)
Grade 3	79 (18.7)	6.2–18.4	12.17 ± 2.19	24 (30.4)
Grade 4	24 (5.7)	5.1–15.9	13.07 ± 2.32	2 (8.3)

ECOG performance score: 0 = fully active; 1 = restricted in physically strenuous activity but able to carry out light work or activities; 2 = ambulatory and capable of self-care but unable to work; 3 = capable of only limited self-care, confined to bed or chair > 50% of time; 4 = completely disabled, totally confined to bed or chair.

**Table 3:** 

<80	Microcytic	<32	Hypochromic
80–100	normocytic	32–36	Normochromic
>100	Macrocytic	>36	Polychromic

*Source:* Taken from Wintrobe's Clinical hematology, 12th edition and McGraw-Hill's Manual of laboratory and diagnostic tests, 2008).

**Table 4:** Relationships between severity of anemia and factors among newly diagnosed solid cancer patients at TASH, Addis Ababa, Ethiopia from April to May 2014 (*n* = 97).

Factors	Severity of anemia				*X * ^2^	*p*-value
	Grade 1	Grade 2	Grade 3	Grade 4		
*Sex*						
Male	14 (56.0%)	7 (8.0%)	2 (8.0%)	2 (8.0%)		
Female	30 (41.7%)	30 (41.7%)	9 (12.5%)	3 (4.2%)	2.609	0.498

*Age (years)*						
18–64	37 (46.2%)	29 (36.2%)	10 (12.5%)	4 (5.0%)		
≥65	7 (41.2%)	8 (47.1%)	1 (5.9%)	1 (5.9%)	1.072	0.829

*Bleeding history*						
NO	31 (59.6%)	14 (26.9%)	5 (9.6%)	2 (3.8%)		
YES	13 (28.9%)	23 (51.1%)	6 (13.3%)	3 (6.7%)	9.387	0.024

**Table 5:** Relationships between prevalence of anemia and factors among newly diagnosed solid cancer patients at TASH, Addis Ababa, Ethiopia from April to May 2014 (*n* = 422).

Variables	Anemia		COR (95% C.I)	*p*-value	AOR (95% C.I)	*p*-value
	Absent	Present				
*Gender*						
Male	119 (82.6%)	25 (17.4%)	1		1	
Female	206 (74.1%)	72 (25.9%)	1.664 (1.001–2.765)^∗^	0.049	1.094 (0.504–2.374)	0.819

*Age (years)*						
18–34	68 (87.2%)	10 (12.8%)	1		1	
35–49	113 (73.9%)	40 (26.1%)	2.407 (1.131–5.124)^∗^	0.023	1.956 (0.845–4.526)	0.117
50–64	105 (77.8%)	30 (22.2%)	1.943 (0.892–4.230)	0.094	1.237 (0.516–2.961)	0.634
≥65	39 (69.6%)	17 (30.4%)	2.964 (1.236–7.108)^∗^	0.015	2.422 (0.925–6.342)	0.072

*Bleeding*						
No	278 (86.1%)	45 (13.9%)	1		1	
Yes	58 (58.6%)	41 (41.4%)	4.343 (2.649–7.121)^∗^	0.001	3.628 (1.800–7.314)^∗^	0.001

*Tumor type*						
Gynecology	76 (62.3%)	46 (37.7%)	3.753 (1.752–8.038)^∗^	0.001	1.444 (0.480–4.346)	0.514
Breast	82 (85.4%)	14 (14.6%)	1.059 (0.441–2.542)	0.899	1.005 (0.355–2.850)	0.992
Colorectal	22 (73.3%)	8 (26.7%)	2.255 (0.790–6.438)	0.129	1.688 (0.544–5.231)	0.365
NPC	24 (74.9%)	8 (25.0%)	2.067 (0.729–5.860)	0.172	2.027 (0.661–6.218)	0.217
Sarcoma	24 (82.8%)	5 (17.2%)	1.292 (0.400–4.172)	0.669	1.470 (0.422–5.124)	0.545
Head and neck	16 (84.1%)	3 (15.9%)	1.162 (0.286–4.725)	0.833	0.849 (0.183–3.936)	0.835
Thyroid	12 (85.7%)	2 (14.3%)	1.033 (0.201–5.323)	0.969	1.234 (0.206–7.408)	0.818
Hepatoma	7 (87.5%)	1 (12.5%)	0.886 (0.098–7.987)	0.914	0.874 (0.086–8.842)	0.909
Others	62 (86.1%)	10 (13.9%)	1		1	

*Tumor stage*						
Stage I	47 (87.0%)	7 (13.0%)	1		1	
Stage II	97 (75.2%)	32 (24.8%)	2.215 (0.911–5.388)	0.08	1.487 (0.564–3.920)	0.423
Stage III	123 (70.7%)	51 (29.3%)	2.784 (1.180–6.569)^∗^	0.019	1.503 (0.565–3.994)	0.414
Stage IV	58 (89.2%)	7 (10.8%)	0.810 (0.265–2.474)	0.712	0.827 (0.240–2.858)	0.764

*ECOG PS*						
Grade 0	67 (19.9%)	11 (12.8%)	1		1	
Grade 1	127 (37.8%)	27 (31.4%)	1.500 (0.726–3.099)	0.273	2.013 (0.918–4.415)	0.081
Grade 2	61 (18.2%)	26 (30.2%)	2.344 (1.088–5.050)^∗^	0.030	3.102 (1.345–7.152)^∗^	0.008
Grade 3	59 (17.6%)	20 (23.3%)	2.400 (1.100–5.235)^∗^	0.028	3.344 (1.410–7.927)^∗^	0.006
Grade 4	22 (6.5%)	2 (2.3%)	0.500 (0.104–2.410)	0.388	0.952 (0.168–5.384)	0.955

Constants are indicated by 1; whereas ^∗^ indicates statistical significant association.

## Data Availability

The survey questioners data used to support the findings of this study are included within the supplementary information file.

## Abbreviations

ACAS: Australian cancer anemia survey
TASH: Tikur Anbessa Specialized Hospital
CBC: Complete blood count
CTCAEV3: Common toxicity criteria for adverse events version 3
ECAS: European anemia cancer survey
ECOG: Eastern cooperative oncology group
EORTC: European organization for research and treatment of cancer
ESA: Erythropoiesis-stimulating agents
HGB: hemoglobin
HCT: Hematocrit
IFN: Interferon
IL: Interleukin
MCV: Mean cell volume
MCHC: Mean cell volume concentration
NPC: Nasopharyngeal carcinoma
RBC: Red blood cell
TNF: Tumor necrosis factor.

## Additional Points

*Limitations*. We have considered lack of conducting iron studies, blood cell morphology evaluation and using cross sectional study design due to budget constraints as our limitations and therefore we have recommended further investigations into the impact of anemia on disease status and treatment outcome using cohort study, iron studies and blood cell morphology evaluation to differentiate microcytic anemia.

## Supplementary Materials

Supplemental Materials The supplementary material is a questionnaire by which we collected data.

## References

[1] Cheng K., Zhao F., Gao F., Dong H., Men H.-T., Chen Y. (2012). Factors potentially associated with chemotherapy-induced anemia in patients with solid cancers. *Asian Pacific Journal of Cancer Prevention*.

[2] Steegmann J. L., Torres J. M. S., Colomer R. (2013). Prevalence and management of anaemia in patients with non-myeloid cancer undergoing systemic therapy: a Spanish survey. *Clinical and Translational Oncology*.

[3] Schrijvers D., De Samblanx H., Roila F. (2010). ESMO Guidelines Working Group: Erythropoiesis-stimulating agents in the treatment of anaemia in cancer patients: ESMO Clinical Practice Guidelines for use. *Annals of Oncology*.

[4] Wan S., Lai Y., Myers R. E. (2013). Post-diagnosis hemoglobin change associates with overall survival of multiple malignancies–results from a 14-year hospital-based cohort of lung, breast, colorectal, and liver cancers. *BMC Cancer*.

[5] Rodgers G. M., Becker P. S., Blinder M. (2012). Cancer- and Chemotherapy-Induced Anemia. *Journal of the National Comprehensive Cancer Network*.

[6] Knight K., Wade S., Balducci L. (2004). Prevalence and outcomes of anemia in cancer: a systematic review of the literature. *The American Journal of Medicine*.

[7] Dicato M., Plawny L., Diederich M. (2010). Anemia in cancer. *Annals of Oncology*.

[8] Kaiser J., Kalyani P., Perimi R., Kameshwari S. V. (2009). Assessment of severity of anemia and its effect on the quality of life (QOL) of patients suffering with various types of neoplasia. *Biology and Medicine*.

[9] Saba H. I. (1998). Anemia in cancer patients: an introduction and overview. *Cancer Control*.

[10] Schwartz R. N. (2007). Anemia in patients with cancer: Incidence, causes, impact, management, and use of treatment guidelines and protocols. *American Journal of Health-System Pharmacy*.

[11] Birgegård G., Aapro M. S., Bokemeyer C. (2005). Cancer-related anemia: pathogenesis, prevalence and treatment. *Oncology*.

[12] Achariyapota V., Benjapibal M., Chaopotong P. (2010). Prevalence and incidence of anemia in Thai patients with gynecologic cancer. *Asian Pacific Journal of Cancer Prevention*.

[13] Aapro M., Österborg A., Gascon P., Ludwig H., Beguin Y. (2012). Prevalence and management of cancer-related anaemia, iron deficiency and the specific role of i.v. iron. *Annals of Oncology*.

[14] Zarychanski R., Houston D. S. (2008). Anemia of chronic disease: a harmful disorder or an adaptive, beneficial response?. *Canadian Medical Association Journal*.

[15] Grotto H. Z. (2008). Anaemia of cancer: an overview of mechanisms involved in its pathogenesis. *Medical Oncology*.

[16] Marchal C., Rangeard L., Brunaud C. (2005). Anaemia impact on treatments of cervical carcinomas. *Cancer Radiotherapie*.

[17] Bahl A., Sharma D. N., Basu J., Rath G. K., Julka P. K. (2008). Pre-Treatment Anemia Evaluation In Cancer Patients Attending Radiotherapy Clinic: Results From A Single Indian Center. *Indian Journal of Medical Sciences*.

[18] Abuzallouf S., Vasishta S., Rageb A., Varghese A., El-Hattab O. (2008). Prognostic value of hemoglobin levels prior to radiotherapy for cervical cancer–Kuwait experience. *The Gulf Journal of Oncology*.

[19] Gao F., Cheng K., Zhao F. (2011). Prevalence and characteristics of anemia in patients with solid cancers at diagnosis in southwest China. *Asian Pacific Journal of Cancer Prevention*.

[20] Barrett-Lee P. (2005). Management of cancer-related anemia in patients with breast or gynecologic cancer: new insights based on results from the european cancer anemia survey. *The Oncologist*.

[21] Hassan F. M., Weeda E. A. (2010). Anemia in elderly sudanese lung cancer patients treated with chemotherapy. *The Open Lung Cancer Journal*.

[22] Ludwig H., Van Belle S., Barrett-Lee P. (2004). The European cancer anemia survey (ECAS): a large, multinational, prospective survey defining the prevalence, incidence, and treatment of anaemia in cancer patients. *European Journal of Cancer*.

[23] Seshadri T., Prince H. M., Bell D. R. (2005). The australian cancer anemia survey: a snapshot of anemia in adult patients with cancer. *Medical Journal of Australia*.

[24] Harrison L., Shasha D., Shiaova L., White C., Ramdeen B., Portenoy R. (2001). Prevalence of anemia in cancer patients undergoing radiation therapy. *Seminars in Oncology*.

[25] Mahasittiwat P., Pataranutraporn P., Ieumwananonthachai N. (2008). Prevalence, incidence and management of anemia in cancer patients treated in the radiation oncology division, Siriraj Hospital. *Siriraj Medical Journal*.

[26] Verbeke N., Beguin Y., Wildiers H. (2012). High prevalence of anemia and limited use of therapy in cancer patients: a Belgian survey (Anaemia Day 2008). *Supportive Care in Cancer*.

[27] Unpublished paper Prevalence of Anemia and Associated Factors Among Newly Diagnosed Patients with Solid Malignancy at Tikur Anbessa Specialized Hospital, Radiotherapy Center, Addis Ababa, Ethiopia. http://etd.aau.edu.et/bitstream/handle/123456789/4343/Edosa%20Kifle.pdf?sequence=1&isAllowed=y.

