# Supplementary material for: Prevalence of Anemia and Associated Factors among Newly Diagnosed Patients with Solid Malignancy at Tikur Anbessa Specialized Hospital, Radiotherapy Center, Addis Ababa, Ethiopia
Source: Adv Hematol. 2019 Oct 20;2019:8279789. doi: 10.1155/2019/8279789 (PMC6855075; doi:10.1155/2019/8279789)
Supplement: Supplemental Materials — The supplementary material is a questionnaire by which we collected data. [file 8279789.f1.pdf]

Annex II: Questionnaire (English version)

INTRODUCTION: Thank you for agreeing to take part in this brief interview. This study is intended to assess the prevalence of anemia and associated factors among newly diagnosed solid cancers patients at TASH, Radiotherapy center, Addis Ababa, Ethiopia. Every data obtained from you will be kept confidential. Without permission from you and legal body, any part of this study will be disclosed to third person. The questionnaire has 3 parts, out of which you belong to one part. It took about 10 minutes to complete the interview. Please try to respond all questions. Thank you very much for your patience.

Part I: Socio-demographic factors questionnaires

S.N	Questionnaires	Alternative response
Q-1	How old are you?	In years, ___________
Q-2	Sex	1. Male<br>2. Female
Q-3	Where is your residence?	1. Rural<br>2. Urban
Q-4	What is your marital status?	1. Single 2. Married<br>1. Divorced 4. Widowed
Q-5	What is level of your education?	1. Illiterate<br>2. Literate
Q-6	What is your job?	1. Employed 2. Merchant<br>3. Farmer 4. Student<br>2. Day laborer 6. House wife

Thank you for your participation!

Part II: Questionnaires for disease and anemia-related factors to be reviewed from medical records

S.N	Questions	Alternative response	
1	Type of tumor	1. Gynecology<br>2. Breast<br>3. Colonic<br>4. Urogenital<br>5. Lung<br>6. Thyroid<br>7. sarcoma	8. anorectal<br>9. Gastric<br>10. NPC<br>11. Head and neck<br>12. Tongue<br>13. Others
2	Stage of cancer	1. Stage I<br>2. Stage II	3. Stage III<br>4. Stage IV
4	Performance status (ECOG)	1. 0<br>2. 1	3. 2<br>4. 3<br>5. 4
5	Is anemia supportive agents ordered for you?	1. YES<br>2. NO<br><br>If yes, which one?	1.1 iron supplement<br>1.2 transfusion<br>1.3 ESA<br>1.4 Others ___________
6	Do you have bleeding history?	1. YES<br>2. NO<br><br>If YES, which one?	1. Hemoptysis<br>2. Haematemesis<br>3.Epistaxis<br>4. Hematuria<br>5. Vaginal Bleeding<br>6. Other

ECOG performance score: 0 = fully active; 1 = restricted in physically strenuous activity but able to carry out light work or activities; 2 = ambulatory and capable of self-care but unable to work; 3 = capable of only limited self-care, confined to bed or chair> 50% of time; 4 = completely disabled, totally confined to bed or chair

Part III: For capturing Laboratory results.

Specimen ID code :		
CBC parameters	Measured value	Reference ranges
RBC count		
Hb		
Hct		
MCV		
MCH		
MCHC		
